# (*E*)-3-Chloro-*N*-[(2-eth­oxy­naphthalen-1-yl)methyl­idene]aniline

**DOI:** 10.1107/S1600536812032114

**Published:** 2012-07-21

**Authors:** Hilal Vesek, Canan Kazak, Ayşen Alaman Ağar, Mustafa Macit, Mustafa Serkan Soylu

**Affiliations:** a Department of Physics, Faculty of Arts and Sciences, Ondokuz Mayıs University, Kurupelit, TR-55139 Samsun, Turkey; b Department of Chemistry, Faculty of Arts and Sciences, Ondokuz Mayıs University, Kurupelit, TR-55139 Samsun, Turkey; c Department of Physics, Giresun University, Arts and Science Faculty, Giresun, Turkey

## Abstract

In the title compound, C_19_H_16_ClNO, the dihedral angle between the naphthalene ring system and the chloro­benzene ring is 61.90 (10)° and the C—N—C—C torsion angle is 174.6 (2)°. The mol­ecular structure is stabilized by an intra­molecular C—H⋯N hydrogen bond. The crystal structure features π–π stacking inter­actions [centroid–centroid distances = 3.7325 (17) and 3.8150 (17) Å].

## Related literature

For applications of Schiff bases in the pharmaceutical industry, medicine, industry and technology, see: Güler (1998 ▶). For their biological properties, see: Lozier *et al.* (1975 ▶); Calligaris *et al.* (1972 ▶); Williams (1972 ▶). For hydrogen-bonding motifs, see: Bernstein *et al.* (1995 ▶). For related structures, see: Zhang (2009 ▶); Pavlović *et al.* (2002 ▶); Özdemir *et al.* (2003 ▶); Inaç *et al.* (2012 ▶); Ağar *et al.* (2010 ▶).
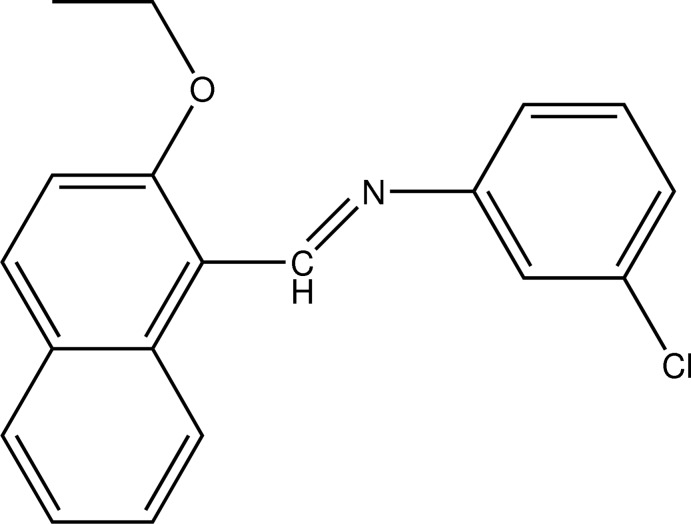


## Experimental

### 

#### Crystal data

C_19_H_16_ClNO
*M* *_r_* = 309.78
Triclinic, 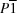

*a* = 8.0084 (14) Å
*b* = 8.7315 (19) Å
*c* = 11.7043 (8) Å
α = 76.253 (13)°
β = 79.794 (10)°
γ = 84.337 (17)°
*V* = 781.0 (2) Å^3^
*Z* = 2
Mo *K*α radiation
μ = 0.25 mm^−1^
*T* = 296 K
0.3 × 0.25 × 0.15 mm

#### Data collection

Stoe IPDS II two-circle diffractometer
Absorption correction: multi-scan (*X-AREA* and *X-RED32*; Stoe & Cie, 2001 ▶) *T*_min_ = 0.793, *T*_max_ = 1.000
5144 measured reflections
3057 independent reflections
2098 reflections with *I* > 2σ(*I*)
*R*_int_ = 0.038

#### Refinement

*R*[*F* ^2^ > 2σ(*F* ^2^)] = 0.058
*wR*(*F* ^2^) = 0.180
*S* = 1.05
3057 reflections
227 parameters
H atoms treated by a mixture of independent and constrained refinement
Δρ_max_ = 0.19 e Å^−3^
Δρ_min_ = −0.32 e Å^−3^

### 

Data collection: *X-AREA* (Stoe & Cie, 2001 ▶); cell refinement: *X-AREA*; data reduction: *X-RED32* (Stoe & Cie, 2001 ▶); program(s) used to solve structure: *SHELXS97* (Sheldrick, 2008 ▶); program(s) used to refine structure: *SHELXL97* (Sheldrick, 2008 ▶); molecular graphics: *ORTEP-3 for Windows* (Farrugia, 1999 ▶); software used to prepare material for publication: *WinGX* (Farrugia, 1999 ▶).

## Supplementary Material

Crystal structure: contains datablock(s) I, global. DOI: 10.1107/S1600536812032114/bx2419sup1.cif

Structure factors: contains datablock(s) I. DOI: 10.1107/S1600536812032114/bx2419Isup2.hkl

Supplementary material file. DOI: 10.1107/S1600536812032114/bx2419Isup3.cml

Additional supplementary materials:  crystallographic information; 3D view; checkCIF report

## Figures and Tables

**Table 1:** Hydrogen-bond geometry (Å, °)

*D*—H⋯*A*	*D*—H	H⋯*A*	*D*⋯*A*	*D*—H⋯*A*
C4—H4⋯N1	0.96 (3)	2.24 (3)	2.915 (3)	127 (2)

**Fig. 1.**
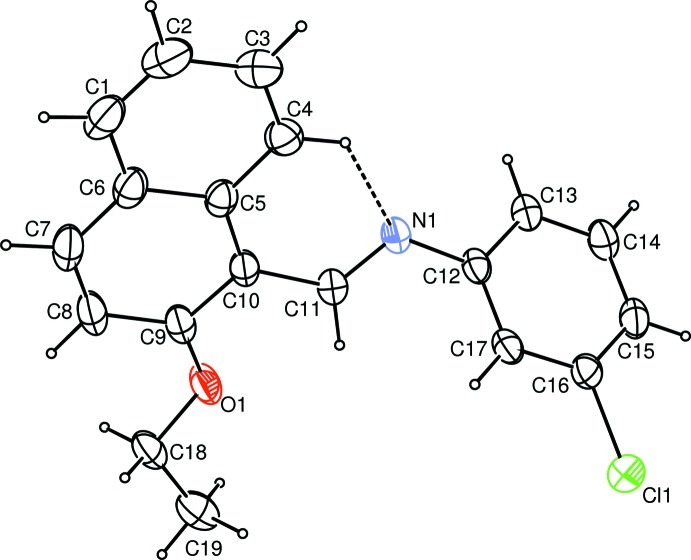
The molecular structure of the title compound, showing the atom-numbering scheme. C—H···N hydrogen bond interaction is shown as dashed lines.

## supplementary crystallographic information

### Comment

Studies with the Schiff bases, started in 1869 to the present and continued intensively. This chemistry is a multi-site and at the same time, lubricants in the pharmaceutical industry, medicine, industry and technology, a wide finds areas (Güler, 1998). Schiff bases are important in diverse fields of chemistry and biochemistry owing to their biological activites (Calligaris *et al.*, 1972; Lozier *et al.*, 1975). Most Schiff bases have antibacterial, anticancer, antinflammatory and antioxic properties (Williams, 1972).As an extension of the study on the structural characterization of Schiff base compounds, the crystal structure of the title compound is reported here. The molecular structure of the title compound are shown in Fig. 1. Bond lengths and angles are normal and comparable with other related compounds (Özdemir *et al.*,2003; (Zhang, 2009; Inaç *et al.*, 2012; Ağar *et al.*, 2010 & Zhang, 2009). The dihedral angle between the naphthalene ring and the chlorobenzene ring is 61.90 (10)°. The molecular structure is stabilized by one intramolecular C—H···N hydrogen bond interaction with S(6) is motif (Bernstein *et al.*, 1995), Table 1. The crystal structure is stabilized by π-π stacking interactions (Cg1–Cg1^i^ = 3.7325 (17) and Cg2–Cg2^ii^ = 3.8150 (17)Å, Cg1 = C5/C6/C7/C8/C9/C10; Cg2 = C12/C13/C14/C15/C16/C17; symmetry codes: (i) -x,-y,-z; (ii) 1-x,-y, 1-z ).

### Experimental

(*E*)-3-chloro-*N*-((2-ethoxynaphthalen-1-yl)methylene)aniline was prepared by reflux of a mixture of a solution containing 2-ethoxy-1-naphthaldehyde (20,0 mg, 0,1 mmol) in ethanol (20 ml) and a solution containing 3-chloroaniline (12,8 mg, 0,1 mmol) in ethanol (20 ml).The reaction mixture was stirred for 5 h under reflux.Single crystals of the title compound for X-ray analysis were obtained by slow evaporation of an ethanol solution (Yield 64%; m.p. 345 - 347 K).

### Refinement

All other H atoms were placed in calculated positions and constrained to ride on their parents atoms, with C—H=0.93–0.96 Å and *U*_iso_(H) = 1.2*U*_eq_(C) or 1.5*U*_eq_(C).

### Crystal data

**Table:**

C_19_H_16_ClNO	*Z* = 2
*M**_r_* = 309.78	*F*(000) = 324
Triclinic, *P*1	*D*_x_ = 1.317 Mg m^−^^3^
Hall symbol: -P 1	Mo *K*α radiation, λ = 0.71073 Å
*a* = 8.0084 (14) Å	Cell parameters from 1666 reflections
*b* = 8.7315 (19) Å	θ = 3.3–28.7°
*c* = 11.7043 (8) Å	µ = 0.25 mm^−^^1^
α = 76.253 (13)°	*T* = 296 K
β = 79.794 (10)°	Prism, yellow
γ = 84.337 (17)°	0.3 × 0.25 × 0.15 mm
*V* = 781.0 (2) Å^3^	

### Data collection

**Table:**

Stoe IPDS II two-circle diffractometer	3057 independent reflections
Radiation source: SuperNova (Mo) X-ray Source	2098 reflections with *I* > 2σ(*I*)
Mirror monochromator	*R*_int_ = 0.038
Detector resolution: 16.0454 pixels mm^-1^	θ_max_ = 26.0°, θ_min_ = 3.3°
ω scans	*h* = −9→9
Absorption correction: multi-scan (*X-AREA* and *X-RED32*; Stoe & Cie, 2001)	*k* = −10→6
*T*_min_ = 0.793, *T*_max_ = 1.000	*l* = −14→14
5144 measured reflections	

### Refinement

**Table:**

Refinement on *F*^2^	Primary atom site location: structure-invariant direct methods
Least-squares matrix: full	Secondary atom site location: difference Fourier map
*R*[*F*^2^ > 2σ(*F*^2^)] = 0.058	Hydrogen site location: inferred from neighbouring sites
*wR*(*F*^2^) = 0.180	H atoms treated by a mixture of independent and constrained refinement
*S* = 1.05	*w* = 1/[σ^2^(*F*_o_^2^) + (0.0836*P*)^2^ + 0.0386*P*] where *P* = (*F*_o_^2^ + 2*F*_c_^2^)/3
3057 reflections	(Δ/σ)_max_ < 0.001
227 parameters	Δρ_max_ = 0.19 e Å^−^^3^
0 restraints	Δρ_min_ = −0.32 e Å^−^^3^

### Special details

**Table:**

Geometry. All e.s.d.'s (except the e.s.d. in the dihedral angle between two l.s. planes) are estimated using the full covariance matrix. The cell e.s.d.'s are taken into account individually in the estimation of e.s.d.'s in distances, angles and torsion angles; correlations between e.s.d.'s in cell parameters are only used when they are defined by crystal symmetry. An approximate (isotropic) treatment of cell e.s.d.'s is used for estimating e.s.d.'s involving l.s. planes.
Refinement. Refinement of *F*^2^ against ALL reflections. The weighted *R*-factor *wR* and goodness of fit *S* are based on *F*^2^, conventional *R*-factors *R* are based on *F*, with *F* set to zero for negative *F*^2^. The threshold expression of *F*^2^ > σ(*F*^2^) is used only for calculating *R*-factors(gt) *etc*. and is not relevant to the choice of reflections for refinement. *R*-factors based on *F*^2^ are statistically about twice as large as those based on *F*, and *R*- factors based on ALL data will be even larger.

### Fractional atomic coordinates and isotropic or equivalent isotropic displacement parameters (Å^2^)

**Table:**

	*x*	*y*	*z*	*U*_iso_*/*U*_eq_	
C1	0.1125 (3)	−0.4097 (4)	0.1075 (3)	0.0711 (9)	
C2	0.1030 (4)	−0.4953 (4)	0.2214 (3)	0.0742 (9)	
C3	0.1347 (4)	−0.4241 (4)	0.3093 (3)	0.0672 (8)	
C4	0.1766 (3)	−0.2695 (3)	0.2822 (2)	0.0556 (6)	
C5	0.1905 (3)	−0.1770 (3)	0.1640 (2)	0.0479 (6)	
C6	0.1568 (3)	−0.2514 (3)	0.0751 (2)	0.0568 (7)	
C7	0.1717 (3)	−0.1636 (4)	−0.0440 (2)	0.0647 (8)	
C8	0.2206 (3)	−0.0141 (4)	−0.0766 (2)	0.0622 (7)	
C9	0.2572 (3)	0.0618 (3)	0.01000 (19)	0.0516 (6)	
C10	0.2407 (3)	−0.0173 (3)	0.12978 (18)	0.0463 (6)	
C11	0.2871 (3)	0.0684 (3)	0.21183 (19)	0.0463 (5)	
C12	0.3167 (3)	0.1180 (3)	0.39163 (17)	0.0443 (5)	
C13	0.2129 (3)	0.1546 (3)	0.49135 (19)	0.0519 (6)	
H13	0.1021	0.1226	0.5121	0.062*	
C14	0.2746 (3)	0.2383 (3)	0.5593 (2)	0.0587 (7)	
H14	0.2037	0.2650	0.6246	0.070*	
C15	0.4401 (3)	0.2833 (3)	0.5319 (2)	0.0570 (6)	
H15	0.4814	0.3395	0.5781	0.068*	
C16	0.5424 (3)	0.2431 (3)	0.43502 (19)	0.0514 (6)	
C17	0.4833 (3)	0.1615 (3)	0.36474 (18)	0.0478 (6)	
H17	0.5549	0.1355	0.2995	0.057*	
C18	0.3509 (3)	0.2904 (3)	−0.13867 (19)	0.0616 (7)	
H18A	0.2530	0.3109	−0.1796	0.074*	
H18B	0.4349	0.2246	−0.1785	0.074*	
C19	0.4234 (4)	0.4418 (4)	−0.1407 (2)	0.0731 (8)	
H19A	0.4575	0.4964	−0.2218	0.110*	
H19B	0.5203	0.4202	−0.1002	0.110*	
H19C	0.3391	0.5062	−0.1015	0.110*	
Cl1	0.75193 (9)	0.29581 (10)	0.40024 (6)	0.0792 (3)	
O1	0.3019 (2)	0.2125 (2)	−0.01705 (13)	0.0651 (5)	
N1	0.2496 (3)	0.0308 (2)	0.32487 (15)	0.0513 (5)	
H1	0.100 (4)	−0.449 (4)	0.045 (3)	0.100 (11)*	
H2	0.076 (4)	−0.609 (5)	0.246 (3)	0.106 (11)*	
H3	0.125 (5)	−0.485 (5)	0.387 (3)	0.128 (14)*	
H4	0.197 (3)	−0.222 (3)	0.344 (2)	0.072 (8)*	
H7	0.154 (4)	−0.209 (4)	−0.107 (2)	0.085 (9)*	
H8	0.232 (3)	0.048 (3)	−0.159 (2)	0.054 (6)*	
H11	0.352 (3)	0.164 (3)	0.1718 (19)	0.048 (6)*	

### Atomic displacement parameters (Å^2^)

**Table:**

	*U*^11^	*U*^22^	*U*^33^	*U*^12^	*U*^13^	*U*^23^
C1	0.0488 (15)	0.087 (2)	0.092 (2)	−0.0112 (14)	−0.0097 (14)	−0.048 (2)
C2	0.0591 (17)	0.066 (2)	0.101 (3)	−0.0156 (14)	0.0025 (15)	−0.0316 (19)
C3	0.0612 (17)	0.0616 (19)	0.0737 (19)	−0.0087 (13)	0.0030 (13)	−0.0132 (16)
C4	0.0558 (15)	0.0559 (17)	0.0557 (15)	−0.0067 (11)	−0.0038 (11)	−0.0158 (13)
C5	0.0385 (12)	0.0591 (16)	0.0510 (13)	0.0016 (10)	−0.0099 (9)	−0.0217 (12)
C6	0.0416 (13)	0.0746 (19)	0.0641 (16)	0.0000 (11)	−0.0107 (10)	−0.0346 (14)
C7	0.0607 (16)	0.088 (2)	0.0599 (16)	0.0032 (14)	−0.0191 (12)	−0.0404 (16)
C8	0.0665 (16)	0.086 (2)	0.0394 (14)	0.0064 (14)	−0.0172 (11)	−0.0224 (14)
C9	0.0552 (14)	0.0638 (17)	0.0378 (12)	0.0032 (11)	−0.0113 (9)	−0.0151 (11)
C10	0.0454 (12)	0.0582 (15)	0.0385 (12)	0.0012 (10)	−0.0105 (9)	−0.0164 (10)
C11	0.0505 (13)	0.0500 (14)	0.0408 (12)	−0.0008 (10)	−0.0113 (9)	−0.0126 (11)
C12	0.0597 (14)	0.0403 (13)	0.0329 (11)	−0.0023 (10)	−0.0128 (9)	−0.0046 (9)
C13	0.0580 (15)	0.0598 (16)	0.0400 (12)	−0.0040 (11)	−0.0113 (10)	−0.0123 (11)
C14	0.0674 (17)	0.0709 (18)	0.0413 (13)	0.0027 (13)	−0.0109 (11)	−0.0205 (12)
C15	0.0750 (17)	0.0586 (16)	0.0436 (13)	−0.0063 (12)	−0.0201 (11)	−0.0151 (12)
C16	0.0622 (15)	0.0508 (15)	0.0407 (12)	−0.0116 (11)	−0.0159 (10)	−0.0005 (11)
C17	0.0593 (14)	0.0496 (14)	0.0325 (11)	−0.0040 (10)	−0.0071 (9)	−0.0050 (10)
C18	0.0633 (16)	0.080 (2)	0.0346 (12)	0.0044 (13)	−0.0065 (10)	−0.0043 (12)
C19	0.0744 (19)	0.077 (2)	0.0536 (16)	0.0060 (15)	−0.0002 (13)	0.0011 (14)
Cl1	0.0712 (5)	0.1066 (7)	0.0619 (5)	−0.0328 (4)	−0.0157 (3)	−0.0082 (4)
O1	0.0954 (14)	0.0659 (13)	0.0335 (9)	−0.0121 (10)	−0.0121 (8)	−0.0060 (8)
N1	0.0615 (12)	0.0582 (13)	0.0373 (10)	−0.0089 (9)	−0.0095 (8)	−0.0136 (9)

### Geometric parameters (Å, º)

**Table:**

C1—C2	1.358 (5)	C11—H11	1.00 (2)
C1—C6	1.407 (4)	C12—C17	1.385 (3)
C1—H1	0.90 (3)	C12—C13	1.391 (3)
C2—C3	1.392 (4)	C12—N1	1.411 (3)
C2—H2	1.00 (4)	C13—C14	1.377 (3)
C3—C4	1.372 (4)	C13—H13	0.9300
C3—H3	0.93 (4)	C14—C15	1.381 (4)
C4—C5	1.418 (3)	C14—H14	0.9300
C4—H4	0.96 (3)	C15—C16	1.372 (3)
C5—C6	1.425 (3)	C15—H15	0.9300
C5—C10	1.434 (3)	C16—C17	1.375 (3)
C6—C7	1.413 (4)	C16—Cl1	1.737 (2)
C7—C8	1.347 (4)	C17—H17	0.9300
C7—H7	0.95 (3)	C18—O1	1.427 (3)
C8—C9	1.420 (3)	C18—C19	1.488 (4)
C8—H8	0.98 (2)	C18—H18A	0.9700
C9—O1	1.347 (3)	C18—H18B	0.9700
C9—C10	1.397 (3)	C19—H19A	0.9600
C10—C11	1.464 (3)	C19—H19B	0.9600
C11—N1	1.273 (3)	C19—H19C	0.9600
			
C2—C1—C6	121.8 (3)	C17—C12—C13	119.2 (2)
C2—C1—H1	124 (2)	C17—C12—N1	122.46 (19)
C6—C1—H1	114 (2)	C13—C12—N1	118.3 (2)
C1—C2—C3	119.3 (3)	C14—C13—C12	119.9 (2)
C1—C2—H2	123.2 (19)	C14—C13—H13	120.0
C3—C2—H2	117.6 (19)	C12—C13—H13	120.0
C4—C3—C2	121.0 (3)	C13—C14—C15	120.9 (2)
C4—C3—H3	122 (2)	C13—C14—H14	119.5
C2—C3—H3	118 (2)	C15—C14—H14	119.5
C3—C4—C5	121.4 (3)	C16—C15—C14	118.6 (2)
C3—C4—H4	120.0 (16)	C16—C15—H15	120.7
C5—C4—H4	118.6 (16)	C14—C15—H15	120.7
C4—C5—C6	116.9 (2)	C15—C16—C17	121.7 (2)
C4—C5—C10	123.8 (2)	C15—C16—Cl1	119.33 (18)
C6—C5—C10	119.2 (2)	C17—C16—Cl1	119.01 (18)
C1—C6—C7	121.9 (2)	C16—C17—C12	119.7 (2)
C1—C6—C5	119.5 (3)	C16—C17—H17	120.2
C7—C6—C5	118.5 (3)	C12—C17—H17	120.2
C8—C7—C6	122.3 (2)	O1—C18—C19	107.9 (2)
C8—C7—H7	116.0 (18)	O1—C18—H18A	110.1
C6—C7—H7	121.6 (18)	C19—C18—H18A	110.1
C7—C8—C9	120.2 (3)	O1—C18—H18B	110.1
C7—C8—H8	123.2 (14)	C19—C18—H18B	110.1
C9—C8—H8	116.6 (14)	H18A—C18—H18B	108.4
O1—C9—C10	117.03 (19)	C18—C19—H19A	109.5
O1—C9—C8	122.7 (2)	C18—C19—H19B	109.5
C10—C9—C8	120.2 (3)	H19A—C19—H19B	109.5
C9—C10—C5	119.4 (2)	C18—C19—H19C	109.5
C9—C10—C11	116.3 (2)	H19A—C19—H19C	109.5
C5—C10—C11	124.1 (2)	H19B—C19—H19C	109.5
N1—C11—C10	125.3 (2)	C9—O1—C18	119.71 (19)
N1—C11—H11	120.4 (12)	C11—N1—C12	118.1 (2)
C10—C11—H11	114.3 (12)		
			
C6—C1—C2—C3	1.2 (4)	C6—C5—C10—C9	−0.6 (3)
C1—C2—C3—C4	−0.5 (4)	C4—C5—C10—C11	1.4 (3)
C2—C3—C4—C5	−0.6 (4)	C6—C5—C10—C11	−176.68 (19)
C3—C4—C5—C6	0.8 (3)	C9—C10—C11—N1	165.2 (2)
C3—C4—C5—C10	−177.3 (2)	C5—C10—C11—N1	−18.7 (4)
C2—C1—C6—C7	178.1 (2)	C17—C12—C13—C14	2.4 (3)
C2—C1—C6—C5	−0.9 (4)	N1—C12—C13—C14	179.4 (2)
C4—C5—C6—C1	−0.1 (3)	C12—C13—C14—C15	−1.8 (4)
C10—C5—C6—C1	178.1 (2)	C13—C14—C15—C16	0.2 (4)
C4—C5—C6—C7	−179.1 (2)	C14—C15—C16—C17	0.7 (4)
C10—C5—C6—C7	−0.9 (3)	C14—C15—C16—Cl1	−179.04 (19)
C1—C6—C7—C8	−177.3 (2)	C15—C16—C17—C12	0.0 (4)
C5—C6—C7—C8	1.7 (4)	Cl1—C16—C17—C12	179.73 (17)
C6—C7—C8—C9	−0.9 (4)	C13—C12—C17—C16	−1.5 (3)
C7—C8—C9—O1	−178.1 (2)	N1—C12—C17—C16	−178.4 (2)
C7—C8—C9—C10	−0.8 (4)	C10—C9—O1—C18	168.6 (2)
O1—C9—C10—C5	178.94 (19)	C8—C9—O1—C18	−14.0 (3)
C8—C9—C10—C5	1.5 (3)	C19—C18—O1—C9	−169.7 (2)
O1—C9—C10—C11	−4.7 (3)	C10—C11—N1—C12	174.6 (2)
C8—C9—C10—C11	177.9 (2)	C17—C12—N1—C11	−42.5 (3)
C4—C5—C10—C9	177.4 (2)	C13—C12—N1—C11	140.6 (2)

### Hydrogen-bond geometry (Å, º)

**Table:**

*D*—H···*A*	*D*—H	H···*A*	*D*···*A*	*D*—H···*A*
C4—H4···N1	0.96 (3)	2.24 (3)	2.915 (3)	127 (2)
